# Continuous wound infiltration or epidural analgesia for pain prevention after hepato-pancreato-biliary surgery within an enhanced recovery program (POP-UP trial): study protocol for a randomized controlled trial

**DOI:** 10.1186/s13063-015-1075-5

**Published:** 2015-12-09

**Authors:** Timothy H. Mungroop, Denise P. Veelo, Olivier R. Busch, Susan van Dieren, Thomas M. van Gulik, Tom M. Karsten, Steve M. de Castro, Marc B. Godfried, Bram Thiel, Markus W. Hollmann, Philipp Lirk, Marc G. Besselink

**Affiliations:** Department of Surgery, Academic Medical Center, University of Amsterdam, Meibergdreef 9, Postbus 22660, 1100 DD Amsterdam, The Netherlands; Department of Anesthesiology, Academic Medical Center, University of Amsterdam, Meibergdreef 9, Postbus 22660, 1100 DD Amsterdam, The Netherlands; Department of Surgery, Onze Lieve Vrouwe Gasthuis, Oosterpark 9, 1091 AC Amsterdam, The Netherlands; Department of Anesthesiology, Onze Lieve Vrouwe Gasthuis, Oosterpark 9, 1091 AC Amsterdam, The Netherlands

**Keywords:** Postoperative pain, Wound catheter, Locoregional analgesia

## Abstract

**Background:**

Postoperative pain prevention is essential for the recovery of surgical patients. Continuous (thoracic) epidural analgesia (CEA) is routinely practiced for major abdominal surgery, but evidence is conflicting on its benefits in this setting. Potential disadvantages of epidural analgesia are a) perioperative hypotension, frequently requiring additional intravenous fluid boluses or prolonged use of vasopressors; b) relatively high failure rates, with periods of inadequate analgesia; and c) the risk of rare but serious, at times persistent, neurologic complications (hematoma and abscess). In recent years, continuous (subfascial) wound infiltration (CWI) plus patient-controlled analgesia (PCA) has been suggested as a safe and reliable alternative, which does not have the previously mentioned disadvantages, but evidence from multicenter trials targeting a specific surgical population is lacking. We hypothesize that CWI+PCA is equally as effective as CEA, without the mentioned disadvantages.

**Methods/design:**

POP-UP is a randomized controlled noninferiority multicenter trial, recruiting adult patients scheduled for elective hepato-pancreato-biliary surgery via laparotomy in an enhanced recovery setting. A total of 102 patients are being randomly allocated to CWI+PCA or (P)CEA. Our primary endpoint is the Overall Benefit of Analgesic Score (OBAS), a composite endpoint of pain intensity, opioid-related adverse effects and patient satisfaction, during postoperative days 1 to 5. Secondary endpoints include length of the hospital stay, number of patients with severe pain, and the use of rescue medication.

**Discussion:**

POP-UP is a pragmatic trial that will provide evidence of whether CWI+PCA is noninferior as compared to (P)CEA after elective hepato-pancreato-biliary surgery via laparotomy in an enhanced recovery setting. If this hypothesis is confirmed, this finding could contribute to more widespread implementation of this technique, especially when the described disadvantages of epidural analgesia are less often observed with CWI+PCA.

**Trial registration:**

Netherlands Trial Register NTR4948 (registry date 2 January 2015).

## Background

Postoperative pain prevention is essential for the recovery of surgical patients. Effective analgesia may prevent complications such as pneumonia, thromboembolic events, and anxiety among patients. Effective analgesia can facilitate early mobilization and hence may result in earlier recovery [1].

Continuous (thoracic) epidural analgesia (CEA) is routinely practiced for pain prevention after elective laparotomy, including hepato-pancreato-biliary (HPB) surgery. This is supported by a recent Cochrane review, which showed statistically but not clinically relevant better analgesia with CEA compared to patient-controlled intravenous analgesia (PCA) alone [2, 3]. Although CEA is sometimes promoted as part of fast-track or enhanced-recovery programs [4], evidence is conflicting on its benefits in this setting [5]. Potential disadvantages of CEA include a) perioperative hypotension, frequently requiring additional intravenous fluid boluses with risk of hypervolemia or prolonged use of vasopressors; b) relatively high failure rates (up to 30 % [6, 7]), with periods of inadequate analgesia; c) the risk of rare but serious and sometimes persistent neurologic complications (hematoma and abscess)(approximately 1 in 5,700 for thoracic epidurals in surgical patients) [8]; and d) the need for indwelling urinary catheters. For these reasons, alternatives have been explored.

Patient-controlled epidural analgesia (PCEA) is rapidly gaining ground. The perceived benefits of PCEA compared to CEA are lower incidences of motor block, smaller infused volume of local anesthetic, and patients being less likely to require unscheduled anesthetic intervention [9]. Not all have switched, however, because PCEA and CEA have a similar safety profile, and randomized trials are lacking.

A promising alternative for (P)CEA is continuous (subfascial) wound infiltration (CWI) plus PCA. In several high volume centers for hepato-pancreato-biliary surgery, CWI+PCA has become the routine analgesic approach for all laparotomies. CWI involves the placement of two to three percutaneous catheters by the surgeon prior to closing of the laparotomy in the preperitoneal space. Continuous infusion of local anesthetics is started via these catheters. This technique potentially lacks the described downsides of (P)CEA. One of the disadvantages of CWI is potentially inferior pain control during the first 24 h requiring PCA with subsequent short-term higher opiate use [10]. Furthermore, CWI+PCA requires the use of two to three extra infusion pumps per patient.

The only randomized controlled trial with hospital stay as primary endpoint concluded that patients fulfilled the discharge criteria faster with the use of wound infiltration compared to epidural analgesia [11].

Although various studies have shown promising results of CWI+PCA [10, 11], a widespread implementation of this technique has not yet been realized. One of the reasons could be the lack of randomized controlled trials, especially trials that focus on specific subsets of abdominal surgical procedures. This study focusses specifically on hepato-pancreato-biliary surgery.

### Objectives

To test the hypothesis that CWI+PCA is noninferior in terms of OBAS compared to (P)CEA for patients undergoing elective hepato-pancreato-biliary surgery via laparotomy with fewer side effects compared to (P)CEA.

### Trial design

This trial is designed as a randomized controlled, parallel group, open-label, noninferiority, multicenter trial.

## Methods/design

### Study design

This is an investigator-initiated randomized controlled noninferiority multicenter trial. The study is being performed in accordance with the Declaration of Helsinki (Fortaleza, Brazil, October 2013) and has been approved by the ethics committee of the Academic Medical Center (AMC) Amsterdam (METC Academisch Medisch Centrum Amsterdam, reference MEC2014_329). Secondary approval was obtained from the board of the Onze Lieve Vrouwe Gasthuis (OLVG) teaching hospital Amsterdam, according to the Dutch Centrale Commissie Mensgebonden Onderzoek (CCMO) External Review Directive 2012 (Richtlijn Externe Toetsing 2012).

Both departments of surgery cooperate regarding hepato-pancreato-biliary surgery within the GastroIntestinal Oncologic Center Amsterdam (GIOCA). Patients are informed about the study on their visit to the outpatient clinic. Afterward, the study coordinator contacts them and provides them the patient information sheet and obtained written informed consent.

### Inclusion/exclusion criteria

Inclusion criteria are as follows: (1) 18 years and older, (2) elective hepato-pancreato-biliary surgery via laparotomy, and (3) written informed consent.

Exclusion criteria are as follows: (1) ASA status ≥ 4, (2) chronic opioid use (>1 year), (3) renal or liver failure, (4) contraindication for epidural analgesia, (5) allergy to study medication, (6) International Normalized Ratio (INR) > 1.5, Partial Thromboplastin Time (PPT) > 1.5, and platelets < 80.

### Randomization

Patients are randomized centrally by the study coordinator using an online randomization module (sealedenvelope.com) in a 1:1 ratio between CWI+PCA and (P)CEA. Randomization is stratified by center and by type of laparotomy (that is, subcostal or midline) to balance differences in general treatment regimens. Blinding of the patient and caregivers is considered infeasible given the obvious differences between the methods (for example, preoperative placement of the epidural). The block size is subject to random variation and concealed to all investigators involved in the study.

### Intervention plan

All patients are treated within an enhanced recovery program. This program consists of (1) preoperative nutritional optimization, (2) absence of preoperative fasting, (3) antithrombotic prophylaxis, (4) early removal of the nasogastric tube, (5) avoiding hypothermia, (6) optimal glycemic control, (7) no drain or early drain removal, (8) maintaining a near-zero fluid balance and avoiding overload of salt and water, and (9) early mobilization.

### Interventional treatment

#### Intervention arm: CWI+PCA

##### Intraoperative

Immediately after laparotomy, the surgeon injects two to three boluses (depending on the type of laparotomy) of 10 ml bupivacaine 0.25 % in the space between peritoneum and posterior fascia at the locations where the two to three CWI catheters are placed at the end of the procedure. At the end of the procedure, wound catheters (standard 18G epidural catheter, REF 4513150, B. Braun Medical, Melsungen, Germany) are placed by the surgeon in the pre-peritoneal space: three in case of a right or left subcostal incision and two in case of a midline laparotomy.

**Fig. 1:**
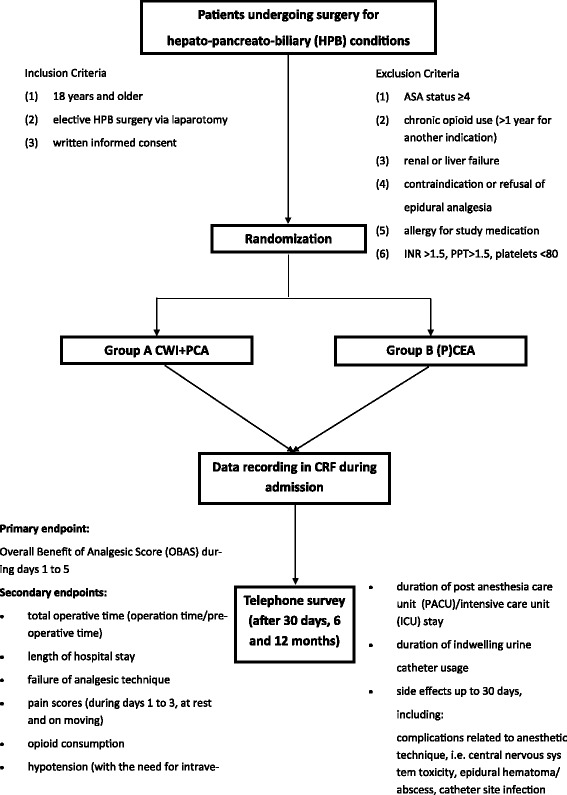
POP-UP flowchart. ASA: american society of anesthesiologists, CWI: continuous wound infiltration, PCA: patientcontrolled analgesia, P(CEA): (patient-controlled) continuous epidural analgesia, INR: internation normalized ratio, PPT: partial thromboplastin time, CRF: case report form

Catheter positions, all in the pre-peritoneal space (see Fig. 1) at the same location in which the boluses were placed at the beginning of the procedure are indicated:

1. Catheter right subcostal region, tunneled via the rectus sheath to the skin.
2. Subcostal under the right rectus muscle sheath, tunneled via the rectus sheath to the skin, exiting close to catheter 1. Catheters 1 and 2 are joined at the filter and stabilized with an adhesive pad. (Statlock, REF IV0621CE, Bard Medical, Nieuwegein, the Netherlands).
3. Subcostal under the left rectus muscle sheath, tunneled via the rectus sheath to the skin, rolled and stabilized with an adhesive pad (Statlock, see 2).

After placement, each catheter is infused with a bolus of 10 ml bupivacaine 0.25 % (total 30 ml).

### Postoperative

The three catheters are each connected to an infusion pump with bupivacaine 0.125 % at 4 ml/hr. In cases of a midline laparotomy, only catheter positions 2 and 3 are used. In this case both run at 6 ml/h of bupivacaine 0.125 %. The maximum combined infusion rate is 12 ml/h over 24 h. The maximum dose of bupivacaine is 0.25 ml/kg. The PCA contains morphine (1 mg bolus with a 5-min lockout). The 4-h maximum dose is 30 ml, equaling 30 mg morphine.

### Control arm: (P)CEA

#### Perioperative

Preoperatively, an anesthesiologist places an epidural catheter at the Th 7 to 8 level. The epidural catheter is used during surgery. The epidural solution consists of bupivacaine (0.125 %) and sufentanil (1 μg/ml). After initial failure of an epidural placement, a maximum of three additional attempts are made. If this fails, this is considered a pre-operative failed epidural, and these patients are then treated according to the intervention arm in order to provide adequate pain management.

### Postoperative

When the treatments are administered continuously at 6 ml/h, patients are able to receive a bolus of 2 ml with a lockout time of 20 min. The 4-h maximum was set at 1.2 mg bupivacaine/kg. In case of a non-patient-controlled epidural (site: OLVG Amsterdam), continuous infusion of a solution (consisting of bupivacaine 0.125 % and sufentanil 0.5 μg/ml) is started (0.1 ml/kg/h).

When CEA or PCEA does not provide adequate pain relief, minor adjustments or a manual top-up are allowed. This is counted as requiring specialist supervision (see Table 1).

**Table 1:** Definitions of technical complications

Failure of placement	Failure of placement (continuous wound infiltration/epidural)
Catheter failure	Catheter dislodgement, migration or leakage, leading to premature removal
Need for rescue medication	Patients remain painful (NRS* > 4) after maximum adjustments possible within the intervention arm
Need for specialist intervention	Suboptimal functioning on ward leading to supervision by an anesthesiologist and/or minor adjustments

*NRS: numeric rating scale

### Adherence to intervention protocols

Participation of patients is communicated to all involved surgery and anesthesiology staff the day before surgery. The implementation of the intervention is followed up by the study coordinator regularly to ensure adherence to the study protocol. Data are checked for completion.

### General treatment regimen

#### Pre-operative

During the study, patients receive standard preoperative care. In addition, induction of anesthesia, blood pressure management, and mechanical ventilation are standard of care and similar between groups.

### Perioperative

#### Induction of anesthesia

In the operating room, general anesthesia is induced with 2 to 3 mg·kg^-1^ propofol (Fresenius Kabi, Zeist, the Netherlands), sufentanil (Bipharma, Almere, the Netherlands) for analgesia and 0.6 mg·kg^-1^ rocuronium (Fresenius Kabi, Zeist, the Netherlands) for paralysis. The trachea is intubated, and the lungs are mechanically ventilated with pressure regulated volume control. After induction, general anesthesia is maintained with sevoflurane (AbbVie, Hoofddorp, the Netherlands) at a minimal alveolar concentration (MAC) of 1 and, when needed (based on signs of pain), is supplemented by an additional bolus of sufentanil. An arterial line is inserted into the right or left radial artery. A right jugular tri-lumen central line is inserted when deemed necessary. A double lumen gastric cannula is inserted. A urinary catheter is inserted. Cefazoline (Kefzol™) 2 g and metronidazol (Flagyl™) 500 mg are given prior to incision. Cefazoline is repeated after 6 h if the surgery is ongoing.

### Analgesia

Besides sufentanil, further analgesia is at the discretion of the anesthesiologist and according to local protocols, which include the use of fentanyl, lidocaine, and ketanest.

### Fluid management

Fluid management is done primarily according to stroke volume (SV), stroke volume variation (SVV), or pulse pressure variation (PPV) guided, goal-directed fluid therapy (GDFT) protocol [12]. SV, cardiac index (CI), PPV/SVV and systemic vascular resistance (SVR) are obtained by means of FloTrac (Edwards Lifesciences) or esophagus Doppler monitoring (EDM). 

### Post-operative

Besides the intervention, patients receive the standard pain treatment with paracetamol (four times 1 g) and metamizol (start with 2 g, followed by four times 1 g, to a maximum of 4 g per day) or naproxen (twice daily 250 mg) (unless contraindicated).

Patients receive a daily visit from the Acute Pain Service (APS), as is a standard of care. The APS team scores the OBAS daily and adjusts the analgesia if necessary. A tentative stop of CWI or (P)CEA is planned on day 3 when feasible and earlier when possible. The APS systematically screens for side effects (for example, monitor sedation levels and puncture sites).

### Rescue medication

If analgesia remains inadequate (NRS > 4) after the maximum adjustments possible within the intervention arm, rescue medication is allowed. This is according to local protocols and includes morphine, piritramide and ketanest.

### Primary endpoint

**Fig. 2:**
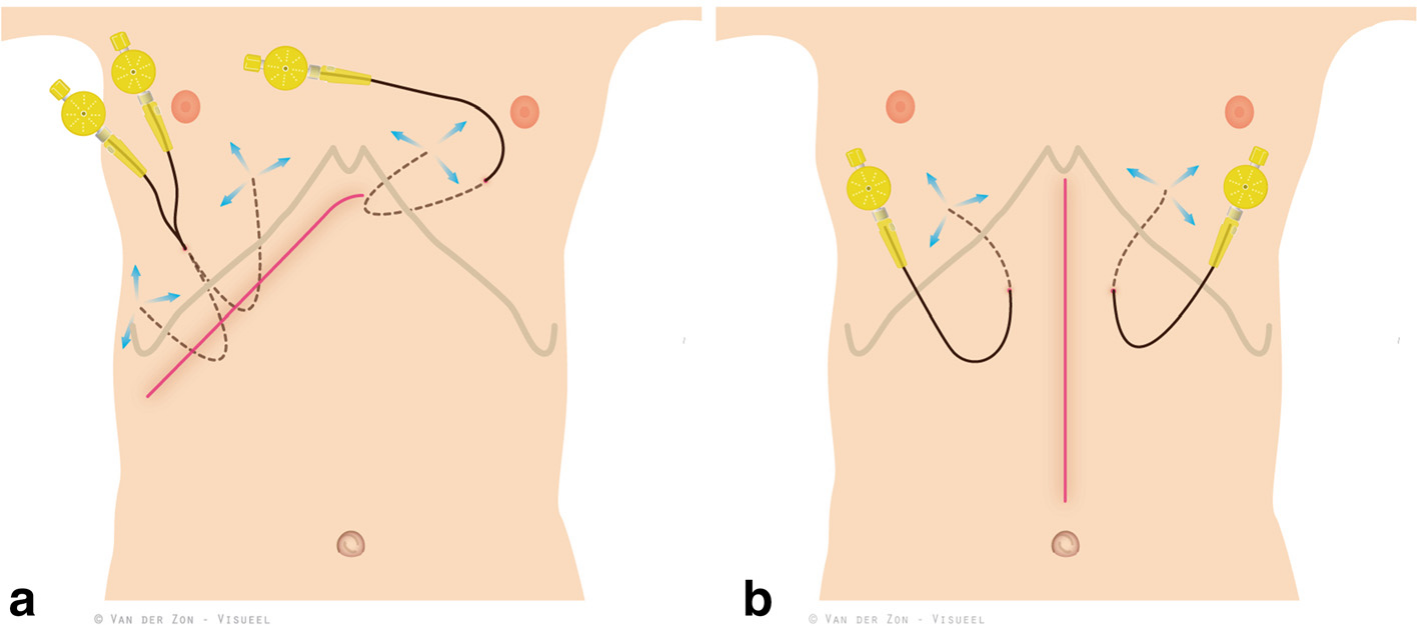
Schematic overview of the location of the CWI catheters in case of subcostal (panel a) and midline incision (panel b)

The primary outcome of this study is the Overall Benefit of Analgesic score (OBAS) (See Fig. 2). The OBAS is a multidimensional quality assessment instrument to measure patients’ benefits from postoperative pain therapy. OBAS is a validated score combining pain score, patient satisfaction and opioid-related side effects [13].

### Secondary endpoints

Secondary endpoints include the total operative time (recorded as total time spent in the operating room, anesthetic time, surgical time); length of hospital stay; failure of analgesic technique; pain every 12 h until discharge at rest and movement (VAS scores); cumulative opioid consumption; additional analgesic consumption; days CWI/PCEA needed; and side effects up to 30 days, including hypotension with the need for additional fluid boluses during and after surgery and norepinephrine dependency. Both at the end of surgery and at the end of the PACU stay, we record the following: 1) duration of norepinephrine requirement and cumulative consumption, 2) fluid boluses administered and 3) fluid balance. The prolonged postanesthesia care unit (PACU)/intensive care unit (ICU) stay; duration of indwelling urinary catheter drainage per day; pruritus; postoperative nausea and vomiting; and complications related to anesthetic technique, such as central nervous system (CNS) toxicity, epidural hematoma, epidural abscess, post-surgical pain (after 30 days, 6 months and 12 months) and analgesics dependency. Technical complications (see Table 2 for definitions) include the following: the failure of analgesic technique, need for rescue medication, and need for specialist intervention.

**Table 2:** Overall Benefit of Analgesic Score calculation sheet [13]. Answers to the following statements are requested from the patient

1. Please rate your current pain at rest on a scale between (0 = minimal pain and 4 = maximum imaginable pain)
2. Please grade any distress and bother from vomiting in the past 24 h (0 = not at all to 4 = very much)
3. Please grade any distress and bother from itching in the past 24 h (0 = not at all to 4 = very much)
4. Please grade any distress and bother from sweating in the past 24 h (0 = not at all to 4 = very much)
5. Please grade any distress and bother from freezing in the past 24 h (0 = not at all to 4 = very much)
6. Please grade any distress and bother from dizziness in the past 24 h (0 = not at all to 4 = very much)
7. How satisfied are you with your pain treatment during the past 24 h (0 = not at all to 4 = very much)

*To calculate the OBAS score, compute the sum of scores in items 1–6 and add “4 − score in item 7”

Overall benefit of analgesia score* |__|__|

### Summary of known and potential risks and benefits

Potential risks of CWI are the need for rescue analgesia, abdominal wound infection and CNS toxicity. Compared to other local anesthetics, bupivacaine is markedly cardiotoxic [14]. However, adverse drug reactions (ADRs) are rare when the bupivacaine is administered correctly.

### Data analysis

The primary analysis will be per protocol. Secondary analyses will include an intention-to-treat analysis. Baseline characteristics will be presented using descriptive statistics. Continuous data will be assessed using a Student’s *t*-test if they are normally distributed or a Mann Whitney *U* test if otherwise. Categorical data will be analyzed using a Chi-square test or Fisher exact test. The mean difference between the two groups will be presented together with the higher bound of the 90 % confidence interval. If, in the per-protocol analysis, the higher bound of the 90 % confidence interval is lower than +3 OBAS point difference and the 90 % confidence interval does not include the inferiority limit, noninferiority is considered proven. Additionally, the difference between the two groups will be assessed with a one-sided Student’s *t*-test or a Mann Whitney *U* test, as appropriate.

Data on the primary and secondary endpoints that are missing will be categorized as no event. For other analyses, data will be considered missing at random. A sensitivity analysis excluding non-patient-controlled-CEA will be performed.

### Sample size

A difference in a mean OBAS score of less than three points is considered noninferior. Therefore, the study must have the power to detect a difference of three points to reject the null hypothesis that CWI is inferior compared to (P)CEA. Using a one-sided alpha of 0.05, a standard deviation of 5 and a power of 90 %, 48 patients are necessary in each group. With an expected loss to follow up of 5 %, the total number of patients to randomize is 102. We aim to complete inclusion within a 12-month period because we have a recruitment pool of 180 laparotomies for hepato-pancreato-biliary conditions per year in both centers combined and expect 60 % of the patients to be both eligible and willing to participate.

### Descriptive statistics

For dichotomous data, frequencies will be presented. Continuous data will be presented as mean and standard deviation or median and interquartile range. Baseline characteristics (all prior to randomization) are as follows: age, sex, body mass index (BMI), American Society of Anesthesiologists (ASA) physical status, smoking/non-smoking, indication for surgery, type of surgery/incision, preoperative pain (as VAS) and use of analgesics.

### Dissemination policy

The trial’s results will be submitted to a peer-reviewed journal regardless of the outcome.

## Discussion

### Benefits

Both CEA and CWI+PCA have been described as effective methods to provide postoperative analgesia after midline or subcostal laparotomy [5, 10, 15] but (P)CEA is current routine practice in most centers worldwide. We hypothesize that the use of CWI+PCA is noninferior to CEA as measured by OBAS and has fewer potential disadvantages than described for CEA. In our opinion, the OBAS score is superior to VAS for evaluating the true effectiveness of analgesic therapy because OBAS is a composite endpoint combining pain score, opiate side effects and patient satisfaction [13]. This is unlike all other previous studies, which focused primarily on pain scores or cumulative opioid consumption. These endpoints are also collected in this study.

A recent large retrospective study suggested equivalent pain scores and a reduced cumulative opioid consumption with the use of the exact same method [10]. Besides that, in a systematic review, different variants of the method have proven to provide a similar analgesic effect compared to CEA measured by pain scores on a visual analog scale [15].

A secondary benefit of CWI+PCA is the broader field of application outside this clinical trial. The technique can be applied in patients with anticoagulant therapy, coagulopathies, and after refusal of epidural analgesia. It may also be used when laparoscopy is converted to laparotomy and in case of emergency surgery. CWI appears to be less invasive in nature and safer because of the absent risk of epidural hematoma and abscess. The placement under general anesthesia can be considered an advantage as well as a disadvantage. When not functioning, replacement is not possible outside the operating room, but an epidural analgesic technique would be possible in that case. Earlier removal of the indwelling urine catheter is possible because there is no additional risk of urinary retention [15]. CWI could require less supervision on the ward by the medical staff [16], although this is heavily dependent on the local situation.

In contrast to other trials, where relatively expensive wound catheters were used that had costs sometimes as much as 280 euro per device [17], the extra cost of the materials used in our study is around 50 euro per patient. This is because we use the standard three-hole epidural catheter instead of the various variations of wound catheters with up to eight or sixteen holes. Since the location is subfascial, diffusion through the whole preperitoneal space is hypothetically possible; therefore, we do not expect advantages with the use of catheters with more side holes. This hypothesis is in line with the results of the previous mentioned retrospective study where the three-hole catheters as well as the same location were used [10]. Our study is the first randomized trial to focus on practice-related benefits like hospital stay, indwelling urinary catheter use, hypotension, PACU/ICU stay and post-surgical pain as well as long-term analgesia dependency.

### Limitations

This is an open-label study. Since patient satisfaction is an important parameter for the OBAS, this design could be of influence. However, since our goal is to pragmatically investigate the entire effect of both treatment strategies and because patients are not blinded in daily practice and multiple placebo catheters (that is, CWI) might influence patient satisfaction, this might be of preference [18]. We chose noninferiority because superiority is not likely to be achieved for the primary outcome parameter. Furthermore, superiority was not considered necessary because of the previously mentioned advantages of CWI+PCA. A limitation of the study is that the patients in one center are treated with non-patient controlled CEA instead of PCEA. We expect this to apply to 20 to 25 % of all recruited patients. A benefit is that we can compare these groups afterwards. A sensitivity analysis will be performed regarding non-patient controlled CEA. One might argue that the addition of PCA to CWI is not necessary. However, previous studies have demonstrated that pain control in the first 24 h after surgery is less with CWI [10], and patients presumably benefit in particular at that time from PCA. In contrast to other studies, we added an additional bolus at the start of the procedure (the bupivacaine bolus of 30 ml total immediately after laparotomy on the final catheter sites). This is meant to compensate for the perioperative use of the epidural that might be the reason for the reduced pain control in the first 24 h. The solution of the epidural consists of local anesthetic combined with an opiate and CWI only provides a local anesthetic. Due to a shortage of morphine receptors in the abdominal layers, the local effect would be negligible and is therefore not indicated in CWI [19]. Therefore, the addition of opiates though PCA might be seen as logical. Another option was to opt for a more pure model by removing sufentanil from the epidural in the control group and also to equip them with PCA. However, because of the pragmatic approach of our trial, as well as our preference to treat the control group according to our current best practice, we chose not to remove sufentanil.

## Trial status

The first patient was randomized on 20 January 2015 with planned completion in February 2016. As of 20 July 2015, 71 of 102 patients (70 %) have been randomized, and inclusion is progressing according to schedule.

## Abbreviations

APS: acute pain service
CEA: continuous (thoracic) epidural analgesia
CWI: continuous (subfascial) wound infiltration
ICU: intensive care unit
OBAS: overall benefit of analgesic score
PACU: postanesthesia care unit
PCA: patient-controlled analgesia
PCEA: patient-controlled epidural analgesia
POP-UP: Continuous wound infiltration or epidural analgesia for pain prevention after hepato-pancreato-biliary surgery within an enhanced recovery program
